# Carbolized Catgut Sutures for Lacerations of the Cervix Uteri

**Published:** 1882-04

**Authors:** A. Reeves Jackson


### CARBOLIZED CATGUT SUTURES FOR LACERATIONS OF THE CERVIX UTERI.

During the past six years I have used as suture material, in the operation for the repair of lacerations of the cervix uteri, silver wire, silk, gut of the silk-worm, and carbolized catgut. As a result of this experience, I can confidently recommend the latter substance as being far superior to either of the others for the purpose indicated.

In my earlier operations I employed silver wire exclusively, and the results were wholly satisfactory so far as maintenance of coaptation of the parts and freedom from irritation were concerned. It had this drawback, however, to its usefulness—a second operation was always necessary for the purpose of removing the stitches. This involved the aid of one or two assistants, the procedure was attended by more or less pain, and was frequently dreaded by the patient almost as greatly as the original operation.

I next tried silk. This I found to be more manageable than wire, and I was enabled by its use to complete the operation more quickly.

With the exception that it produced some slight and unimportant ulceration at the places of the sutures, it gave equally good results with the wire, and its removal was attended by less pain.

But I wanted something that would obviate the need for the secondary use of scissors and forceps, and in my quest I was deluded by the beauty of the silk-worm gut, and the hope that, being an animal substance, it might dissolve and disappear without assistance. But it did not. In one instance I permitted it to remain nearly four weeks, at the end of which time it appeared to have undergone no change, and I removed it as though it were wire. It was as easily adjusted as silk, and as capable of being firmly knotted, while, at the same time, it produced no more irritation than wire ; but it presented no material advantage over either of those substances.

Finally, I ventured to try catgut ; not wholly, at first, for I feared that, from its well-known solubility when imbedded in the tissues and fluids of the body, it might not retain the parts in apposition sufficiently long for union to take place. I therefore placed the animal sutures alternately with others of wire. On removing the latter, on the tenth day after operation, I could find no trace of the gut threads. Union was perfect. Subsequently, in another case, I used wire on one side and catgut on the other. On the eighth day following, I discovered only a few softened shreds of the latter. The union of the parts was equally complete on either side, and there was less appearance of redness on the side which had been closed with the catgut than on the other in which silver was used.

Latterly—for more than a year—I have been using catgut almost exclusively ; and in no instance has it failed to hold the denuded surfaces together until firm adhesion has occurred. Having performed this function, it melts away and disappears, giving no trouble either to patient or surgeon.

But, in order to obtain such desirable result, I would caution the inexperienced operator that great care must be taken in paring the flaps. This must be done in such direction, and to such extent, as to permit the opposing surfaces to be brought *very easily* together ; otherwise, the resulting swelling will cause any suture to "cut" more or less, and failure may ensue.

By immersing the catgut in warm water a few minutes before using it, it becomes almost as pliable as silk and can be as readily tied. I always make a triple knot.

While I consider the use of catgut as superior for the suture to either wire or silk in all ordinary cases of lacerated cervix, it is especially indicated in those in which repair of the torn perineum is likewise required; for both operations can then be done at the same time without making occasion for jeopardizing the new perineal adhesions by putting them on the stretch for the purpose of removing the stitches from the cervix. In the use of wire or silk we must either do this, or accept the alternative of waiting several weeks for the perineal structures to safely bear such strain; a delay obviously objectionable, especially to the patient.—*Dr. A. Reeves Jackson, in Medical Record.*

